# Tensile Strength of the Achilles Tendon Allograft: A Comparative Study of Graft Preparation Technique

**DOI:** 10.3390/jcm13216488

**Published:** 2024-10-29

**Authors:** Grace E. Thiel, Tyler D. Perleberg, Troy B. Puga, Benedict F. Figuerres, Ganesh Thiagarajan, Jennifer F. Dennis

**Affiliations:** 1 College of Osteopathic Medicine, Kansas City University, Kansas City, MO 64106, USAtyler.perleberg@kansascity.edu (T.D.P.);; 2 Department of Orthopedics and Sports Medicine, University of Kansas Health System St. Francis Campus, Topeka, KS 66615, USA; 3 Department of Biomedical Engineering, School of Science and Engineering, University of Missouri, Kansas City, MO 64112, USA; ganesht@umkc.edu; 4 Department of Academic Affairs, Kansas Health Science Center, Kansas College of Osteopathic Medicine, Wichita, KS 67202, USA

**Keywords:** Achilles tendon, allograft, rotational fibers, anterior cruciate ligament reconstruction

## Abstract

**Background/Objectives**: The Achilles tendon is a popular allograft option for anterior cruciate ligament (ACL) reconstruction. Structurally, the tendon is known to have a 90-degree rotational fiber track. Preparation techniques, with this consideration, may influence the strength of the graft. This study aims to assess the tensile strength of a novel Achilles tendon allograft harvest procedure following the rotational fiber track. **Methods**: Both Achilles tendons were harvested from formalin-embalmed cadavers [(*n* = 20), male *n* = 13, female *n* = 7, average age = 70]. Ten cadavers had the right Achilles as the control and the left Achilles as the fiber track sample; 10 cadavers had the opposing designation. Tensile strength was tested utilizing a Bose machine. An unpaired t-test was used to compare data across groups. **Results**: The average ultimate load for the control group was 874.17 N, with an average elastic stiffness of 76.01 N/mm. The ultimate load for the fiber track group was 807.84 N, with an average elastic stiffness of 64.75 N/mm. No statistically significant difference (*p* = 0.21) was determined between the average ultimate loads or elastic loads (*p* = 0.18) across groups. **Conclusions:** These data suggest that the rotational fiber track method of Achilles allograft has consistent tensile strength and elastic stiffness as compared to the common harvest procedure. The rotational fiber track method for ACL harvesting is a viable alternative option to the common harvest procedure for usage in an ACL reconstruction.

## 1. Introduction

Anterior cruciate ligament (ACL) injuries are one of the most common sources of knee injuries in the world and appear to be on the rise in both adults and adolescents [1,2]. While non-operative management is a valid option for some patients and results in the yield of similar long-term results in quadriceps strength [3], operative reconstructions are frequently performed to reduce the need for secondary meniscectomies and to provide the patient with better knee function [2,4]. ACL reconstruction (ACL-R) is an established operative procedure to address and correct a torn ACL [2,4,5]. Importantly, a primary ACL-R procedure can fail, often resulting in the need for a revision ACL-R. The average revision rate is 2.9–5.8% in the general population, and revision is often necessitated after graft rupture or laxity [6]. Therefore, factors such as graft selection, technique, rehabilitation, and patient characteristics are important considerations for any reconstruction [2,4,7,8,9,10,11], with graft selection having been suggested as one of the more important considerations regarding the success of both primary and revision ACL-R procedures [5,6].

When it comes to both primary and revision ACL-Rs, there are a variety of graft options including both autografts and allografts. Allograft utilization has increased over the years, especially in older patients [7,8,9,10,12]. Furthermore, allografts provide several advantages over autografts, including a predictable and modifiable graft size, a lack of harvest site morbidity, and decreased surgical times [7]. The use of the Achilles tendon as an allograft has become an increasingly popular option with positive outcomes reported for both primary and revision ACL-Rs [8,10,12,13]. Yet, the preparation for Achilles tendon allografts is not well detailed in the literature, as there is a lack of clarification regarding which aspect of the tendon is best to utilize. One harvest technique in the literature gives no advice or input regarding the portion of the tendon, abut to the calcaneus, that should be used [14]. Instead, the technique simply suggests that the harvest procedure should begin with the alteration of the width, length, and thickness of the bone end of the tendon graft to form a symmetrical bone block [14]. This technique also includes longitudinal dissection of the tendon portion of the graft, which is made in line with the previously measured width of the bone block [14].

With the wide variety of graft options for an ACL-R, various studies have been conducted to assess the quality of the graft. One such testing method is through tensile strength assessment. As such, a variety of different grafts for an ACL-R procedure, such as hamstring tendons and quadriceps tendons, have been assessed regarding the tensile strength [15]. Previous studies have assessed the tensile strength of the Achilles tendon and the Achilles tendon as an allograft, but these studies either utilized the entire tendon or remnants from it after it was harvested for use as an ACL-R allograft [4,11]. Weber et al. [16] even went as far as to state that “further work is necessary to evaluate the regional significance of Achilles tendon allografts” when discussing their own usage of the outer portion of the tendon for tensile strength assessment [16]. Collectively, this does not represent the true quality expected for an allograft, as allografts are prepared from a central aspect of the Achilles. Further, no previous studies have regarded the 90-degree rotational fiber track of the Achilles tendon [16,17,18,19], which was an important consideration for the authors.

Recent work characterized an Achilles tendon allograft preparation utilizing the rotation of the fibers of the Achilles tendon for ACL-R [20]; however, the tensile strength of the rotational allograft was not evaluated. Building on this report, our objective was to compare the tensile strength of two different Achilles tendon allograft preparation techniques. It was hypothesized that Achilles tendon allografts harvested with attention to the rotational fibers, as opposed to straight blunt cuts through the rotational fibers, would produce a greater tensile strength.

## 2. Materials and Methods

### 2.1. Cadaveric Material

Twenty-seven formalin-embalmed cadavers were included in the original study population. All subjects gave their informed consent for inclusion before they participated in the study. The study was conducted in accordance with the Declaration of Helsinki, and the protocol was approved by the Institutional Biosafety Committee (#1981117-1). The Achilles tendons were dissected out of the donors bilaterally. This was achieved by first exposing the tendon, gastrocnemius, soleus, and calcaneus. The muscle bellies of the gastrocnemius and soleus were removed from the cadaveric donor to produce excess tendon and muscle fibers that would later be removed. A large bone block was created from the calcaneus, as the bone was cut from lateral to medial with an oscillating saw. The specimens, now free from the donors, were further cleaned to resemble the expected quality of an allograft delivered to an operating room for an ACL-R. At this time, all specimens were closely examined for damage. If one of the tendons appeared to have any damage, both tendons from the donor were excluded from the study. Seven of the donors were excluded during this process. Therefore, the resulting final study population was 20 cadavers (*n* = 20; 13 males, 7 females; average age = 70).

### 2.2. Experimental Design

Two study populations were created: the control group, which was harvested with no regard to the fiber track, and the fiber track group, which was harvested with special attention given to the fiber track to preserve as many fibers as possible. Each of the 20 donors were assigned a number. These numbers were placed in a random number generator with 10 numbers being selected. These 10 randomly selected donors were chosen to have their right Achilles designated as the control group, meaning that their left Achilles would be placed in the fiber track group. The remaining 10 donors had the opposing designation with the left Achilles being in the control group and the right Achilles in the fiber track group. This was done to help minimize any possible bias that might have occurred if the specimens were in view while deciding the study group distinction, and to help account for any differences in tendon properties of right versus left.

Once the study group was determined, the specimens were prepared for biomechanical testing. The bone block was removed at the tendon’s insertion into the calcaneus to aid in the biomechanical testing process. This step was decided based on the pretest and gripping issues, such as slipping, with the bone block. The control group’s tendons were cut with a 10 mm width, with no regard to fiber direction. Conversely, the fiber track group required close examination to determine the direction of the fiber track, as can be visualized grossly. The approach for isolating the rotational fiber track has been previously reported by our group [20]; briefly, the allograft was evaluated with special attention to the rotational fiber track. The rotational fiber track was identified and marked using a sterile, latex-free–tip surgical marker to permit enough room for the 10 mm width requirement (for the bone block). Careful dissection was performed following the markings (10 mm width) to remove the excess tendon. This method produced the two different sample groups that would later be biomechanically tested.

### 2.3. Biomechanical Testing

**Figure 1:**
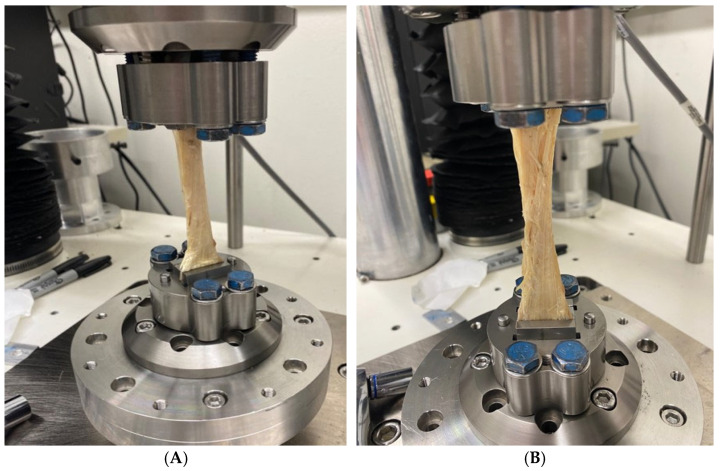
(**A**) Tensile strength testing set-up with allograft sample. The tissue sample vertically positioned in the Bose machine is depicted. The two free tendon ends were wrapped in gauze and placed in the superior or inferior grips. The end of the tendon that was previously attached to the bone block was placed in the superior grip. Slack was removed from the sample and a force was placed on the sample at a rate of 0.25 mm/s until a peak force was reached. The test was stopped when either the tendon failed or the load dropped well beyond the peak load. (**B**) Image of allograft sample rupture due to peak, as indicated by tears and fraying in tissue sample.

Upon testing, the width and thickness of the tendon were measured, and the exact width was remeasured (Table 1); all measurements were performed with a Vernier Digital Caliper (Mitutoyo, Sakado, Takatsu-ku, Kawasaki, Kanagawa 213-8533, Japan). The tendons were individually tested in the same manner, regardless of sample group. The two free tendon ends were wrapped in gauze and placed in a superior or inferior grip. The end of the tendon that was previously attached to the bone block was placed in the superior grip in the vertical setup of the Bose 3510-AT machine, (TA Instruments Electroforce, Eden Prairie, MN, USA).

(Figure 1A). Slack was removed from the sample and a force was placed at a rate of 0.25 mm/s until a peak force was reached. The test was stopped when either the tendon failed or the load dropped well beyond the peak load. The study utilized a single load-to-failure protocol (Figure 1B).

**Figure 2:**
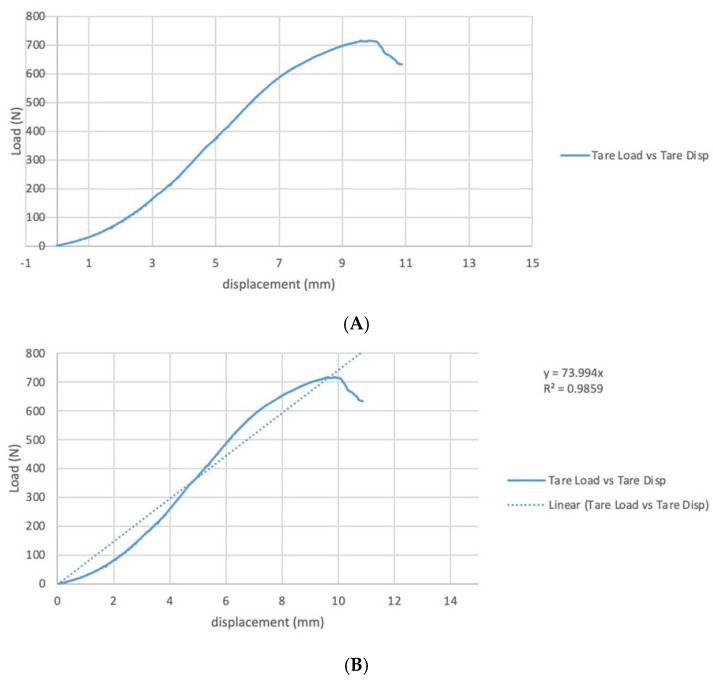
(**A**) Sample load displacement curve as measured using the Wintest Software (Version 7, (TA Instruments Electroforce, Eden Prairie, MN, USA). The software records the data throughout the testing process. Outcomes that were calculated for comparison were the initial stiffness of the load displacement curve, the ultimate load, and the ultimate stress of the tendons. (**B**) Graph of the slope of the load displacement curve. The elastic stiffness was taken as the slope of the load–displacement curve, which represents the stiffness of the tendon as it responds to normal physiological loading (same sample as depicted in Figure 1).

The force and deformation were measured using the Wintest Software (Version 7, (TA Instruments Electroforce, Eden Prairie, MN, USA) that comes with the Bose testing machine. This software recorded the data throughout the testing process. The location of the tear was noted before moving on to the next specimen (Figure 1B). Data from each test were later exported to a Microsoft Excel file for analysis (Microsoft Corp, Redmond WA). A sample load–displacement curve was created, as is shown in Figure 2A. Outcomes that were calculated for comparison were the initial stiffness of the load–displacement curve, the ultimate load, and the ultimate stress of the tendons. The elastic stiffness was taken as the slope of the load–displacement curve, which represents the stiffness of the tendon as it responds to normal physiological loading (Figure 2B). The ultimate load (in Newtons), while structurally important, would only be comparable if all the specimens have the same cross-sectional area. This was not possible to achieve in the current experimental setup. However, one metric that is comparable across all specimens is the ultimate stress, which is defined as the ultimate load divided by the cross-sectional area of the specimen. In addition, the elastic stiffness divided by the cross-sectional area was considered to normalize the slope for comparison across all specimens.

### 2.4. Data Analysis

A two-sample equal variances two-tailed t-test was used to compare the data between the control group and the fiber track group for all metrics. The following metrics were analyzed with the two-sample equal variances two-tailed t-test: mean ultimate load, mean elastic stiffness, ultimate load per width, ultimate load per area (or ultimate stress), elastic stiffness per width, and elastic stiffness per area. All analyses were conducted in Microsoft Excel (Version 16.58, Microsoft Corporation, Redmond, WA, USA). An alpha value of 0.05 was utilized for the study.

## 3. Results

The control group had a mean ultimate load of 874.17 N and the fiber track group had a mean ultimate load of 807.84 N (*p* = 0.21). The control group had a mean elastic stiffness of 76.01 N/mm and the fiber track group had a mean elastic stiffness of 64.75 N/mm (*p* = 0.18). The control group had a mean ultimate load per width of 89.45 N/mm and the fiber track group had a mean ultimate load per width of 82.79 N/mm (*p* = 0.25). The control group had a mean ultimate stress of 15.73 N/mm^2^ and the fiber track group had a mean ultimate stress of 16.45 N/mm^2^ (*p* = 0.61). The control group had a mean elastic stiffness per width of 7.84 N/mm/mm and the fiber track group had a mean elastic stiffness per width of 6.73 N/mm/mm (*p* = 0.26). The control group had a mean elastic stiffness per area of 1.40 N/mm/mm^2^ and the fiber track group had a mean elastic stiffness per area of 1.33 N/mm/mm^2^ (*p* = 0.74) (Table 2).

## 4. Discussion

This study did not result in any significant findings regarding the tensile strength properties of the fiber track group when compared to the control group. However, it is interesting to note that the mean elastic stiffness of the fiber track group was lower compared to the control specimens. This might indicate that the fiber track harvest method resulted in greater stiffness, although it was statistically insignificant. The fiber track group also showed a higher mean ultimate stress compared to the control group. Again, while this was statistically insignificant, the associated difference might suggest the possibility for a clinical difference between study groups, which could impact patient outcomes.

Previous studies have examined the tensile strength of the Achilles tendon and the Achilles tendon as an allograft [16,18,21]. Initial characterization of tensile strength and cross-sectional area by Louis-Ugbo and colleagues in fresh Achilles tendons noted a statistical difference in the cross-sectional area of the Achilles tendon when evaluating laterality, but no differences in the tensile strength across the limbs [18]. Additionally, the specimens evaluated were not prepared in a manner consistent with current allograft techniques. More recent characterization of biomechanical properties of 19 tendons and ligaments in fresh-frozen cadavers has revealed a statistically significant difference in the Achilles tendon failure load compared to others evaluated, characterized with the second-highest force in Newtons to failure, only behind quadriceps tendon (intact) [21]. In contrast, it has significantly lower failure strain (elastic modulus) compared to other ankle and hamstring tendons, indicating a decreased ability of the tissue to withstand higher forces and resist tissue deformation [21]. Of note, all specimens analyzed were retrieved by an orthopedic surgeon to ensure consistency of the samples; however, the Achilles tendon samples were not prepared with reference to fiber track orientation [21]. Finally, cyclic biomechanical evaluation of frozen and gamma-irradiated Achilles tendon specimens revealed an average tendon elongation (also known as creep) of 1.4% ± 1.6% with 100 cycles at 1 Hz [16]. Reported values for maximum stress and modulus (stiffness) in these specimens are difficult to compare across studies as the load failure was not analyzed, only modulus. Like previous reports, the biomechanical properties reported for the Achilles specimens were not impacted by donor age or location of graft failure [16].

This study presented the first examination of the tensile strength of a novel harvest technique. This technique relies on close inspection of the gross rotational fiber tracks within the tendon, allowing for fiber preservation during the harvesting procedure. The lack of significant difference in tensile strength between the techniques demonstrates that the fiber track harvest method is neither superior nor inferior to the common graft preparation technique; however, the fiber track harvest method does provide an alternative option for surgeons when preparing an Achilles tendon for use as an allograft. Indeed, surgeons might choose to utilize this method as they may feel more comfortable ensuring that tendon fibers are not unnecessarily disturbed. It would be of value to complete a comparison evaluation of the biomechanical properties of standard and rotational fiber track allografts in fresh and/or fresh-frozen donors, including cyclical testing, to better appreciate any differences in tissue behavior specific to ACL reconstruction. Indeed, this would complete a current gap in the literature specific to reports on the characterization of the Achilles rotational fiber track.

With the high frequency of ACL injuries in young athletes and those involved in collision sports, ACL-R remains a crucial treatment for those wishing to return to athletic activity and normal functional capacity [1,17,22,23,24,25]. Young athletes also typically face a higher revision rate over the general population, although the risk of re-rupture is possible in all cases [6]. Therefore, regardless of the population, careful consideration should be granted towards graft selection [2,4,26]. The Achilles tendon offers a valid option for ACL-R in both primary and revision cases [5,6]. Additionally, an Achilles tendon allograft can be utilized in other reconstructive procedures, such as in the case of both primary and revision PCL reconstructions [18,27].

### 4.1. Limitations

This study was not without limitations. The study utilized formalin-embalmed cadavers as a source of the study specimens. Fresh-frozen cadavers and formalin-embalmed cadavers have been used to test the viability of a variety of different ACL grafts [16,28,29,30]. While formalin-embalmed grafts may have stiffer properties and decreased stretch when compared to fresh-frozen grafts, they can still provide favorable results and comparable load-to-failure results as fresh-frozen grafts [28,30]. Although there is still an on-going debate about which type of cadaveric graft should be utilized in tensile strength assessment, both formalin-embalmed and fresh-frozen types have been shown to be effective within research methods. However, given the results of this study with statistically insignificant differences in elastic properties between study groups, fresh-frozen grafts may portray more accurate findings.

Additionally, this study only assessed one study design regarding tensile strength. The current study testing protocol involved a constant rate of increasing force until failure. There are testing designs that would consider a variety of patient scenarios such as an assessment of fatigue and a comparison of plastic versus elastic deformation. Future studies should consider these important characteristics for a more complete understanding of the two allograft groups within this study.

Finally, the current study consisted of a population of donors (mean age, 70 years) with known comorbidities and/or physical limitations that could have impacted tendon quality. To account for these factors, one sample per donor was placed in each study group, resulting in only differences between the preparation technique itself and any extremity-specific differences. Despite this, not all comorbidities could have been accounted for in the given donor population, as a donor might have had an undocumented unilateral limitation.

### 4.2. Next Steps

Being that the tensile strength is comparable, follow-up studies should examine the tensile strength of these two study groups utilizing fresh-frozen specimens, with special regards to stiffness differences. Other studies should also look to examine if there is a significant clinical difference when using the fiber track harvest method of allograft preparation compared to other common harvest methods. While a consensus regarding the optimal graft and graft preparation may never be reached, it is important that testing and outcome studies are continued regarding graft selection to help best guide surgeons when performing an ACL-R. Additional surgical consideration, such as surgical techniques and patient characteristics, should also be considered in ACL-R [1,7,9]. Further large-scale studies should aim to compare the combination of techniques, patient characteristics, and graft selection for patient outcomes, as there could be particular patterns that may lead to optimal success.

## 5. Conclusions

The fiber track harvest method for Achilles allograft preparation is neither superior nor inferior to the common graft preparation method based on the comparison of tensile strengths, as there was no difference found between the two harvest methods. Therefore, the fiber track harvest method may provide a reasonable alternative to other graft preparation techniques that can be used based upon surgeon preferences for ACL-R graft preparation.

## Figures and Tables

**Table 1:** Donor specimen characteristics.

	Width	Thickness	Cross-Sectional Area
Mean	9.83 mm	5.50 mm	54.10 mm^2^
Maximum	11.49 mm	7.45 mm	77.18 mm^2^
Minimum	8.19 mm	4.13 mm	33.82 mm^2^

**Table 2:** Means and standard deviations of study variables.

Variable	Control Mean (SD)	Fiber Track Group Mean (SD)	*p*-Value
Ultimate load	874.17 N (176.63)	807.84 N (148.61)	0.21
Elastic stiffness	76.01 N/mm (33.83)	64.75 N/mm (15.46)	0.18
Ultimate load/width	89.45 N/mm (20.35)	82.79 N/mm (15.16)	0.25
Ultimate load/area	15.73 N/mm^2^ (4.98)	16.45 N/mm^2^ (3.68)	0.61
Elastic stiffness/width	7.84 N/mm/mm (3.89)	6.73 N/mm/mm (1.95)	0.26
Elastic stiffness/area	1.40 N/mm/mm^2^ (0.80)	1.33 N/mm/mm^2^ (0.42)	0.74

## Data Availability

The original contributions presented in the study are included in the article, and further inquiries can be directed to the corresponding author/s.
